# Identification and characterization of pineapple leaf lncRNAs in crassulacean acid metabolism (CAM) photosynthesis pathway

**DOI:** 10.1038/s41598-019-43088-8

**Published:** 2019-04-30

**Authors:** Youhuang Bai, Xiaozhuan Dai, Yi Li, Lulu Wang, Weimin Li, Yanhui Liu, Yan Cheng, Yuan Qin

**Affiliations:** 1 College of life science, Fujian Agriculture and Forestry University, Fuzhou, 350002 China; 2 State Key Laboratory for Conservation and Utilization of Subtropical Agro-Bioresources, Guangxi Key Lab of Sugarcane Biology, College of Agriculture, Guangxi University, Nanning, 530004 Guangxi China; 3 College of Resources and Environment, Fujian Agriculture and Forestry University, Fuzhou, 350002 China; 4 College of Plant Protection, Fujian Agriculture and Forestry University, Fuzhou, 350002 China

**Keywords:** Photosynthesis, Plant sciences

## Abstract

Long noncoding RNAs (lncRNAs) have been identified in many mammals and plants and are known to play crucial roles in multiple biological processes. Pineapple is an important tropical fruit and a good model for studying the plant evolutionary adaptation to the dry environment and the crassulacean acid metabolism (CAM) photosynthesis strategy; however, the lncRNAs involved in CAM pathway remain poorly characterized. Here, we analyzed the available RNA-seq data sets derived from 26 pineapple leaf samples at 13 time points and identified 2,888 leaf lncRNAs, including 2,046 long intergenic noncoding RNAs (lincRNAs) and 842 long noncoding natural antisense transcripts (lncNATs). Pineapple leaf lncRNAs are expressed in a highly tissue-specific manner. Co-expression analysis of leaf lncRNA and mRNA revealed that leaf lncRNAs are preferentially associated with photosynthesis genes. We further identified leaf lncRNAs that potentially function as competing endogenous RNAs (ceRNAs) of two CAM photosynthesis pathway genes, *PPCK* and *PEPC*, and revealed their diurnal expression pattern in leaves. Moreover, we found that 48% of lncRNAs exhibit diurnal expression patterns in leaves, suggesting their important roles in CAM. This study conducted a comprehensive genome-wide analysis of leaf lncRNAs and identified their role in gene expression regulation of the CAM photosynthesis pathway in pineapple.

## Introduction

Over the past decade, an increasing number of long (>200 nt) noncoding RNAs (lncRNAs) have been identified by large-scale genomic studies. Recent developments in RNA sequencing technology (RNA-seq) and computational methodology have made it possible to identify and characterize these lncRNAs from short read RNA-seq data. Besides the considerable number of lncRNAs that have been identified in model plant organisms, such as Arabidopsis^1–4^, Rice^5^, and maize^6^, plenty of non-model plants have revealed more novel lncRNAs, such as *Medicago truncatula*^7^ and wheat^8^, peach^9^, Populus^10,11^, soybean^12^, and *B. rapa*^13^. Many lncRNAs function in diverse biological processes, like gene silencing, responses to abiotic or biotic stress, RNA alternative splicing, translational control, reproduction, and chromatin modification^4,5,14–17^. A hypothesis for competing endogenous RNA (ceRNA) proposed that lncRNAs, circular RNAs (circRNAs), mRNAs, and other types of RNAs can function as natural miRNA sponges to inhibit normal miRNAs targeting activity on mRNA by sharing common miRNA responsive elements (MREs)^18^. This hypothesis that lncRNA acted as ceRNA to regulate mRNAs expression through competing for common miRNAs has been validated experimentally by previous studies^19^. LncRNAs functioned as ceRNA competes for available miRNA in cells, which can sequester miRNAs away from their targets. More importantly, newly identified intricate ceRNA networks will promote the understanding of the language of lncRNA-mediated ceRNA regulatory mechanisms.

CAM (crassulacean acid metabolism), also known as CAM photosynthesis, is an efficient pathway for some plants, such as pineapple, to survive in arid environments^20,21^. CAM differs between the C3 and C4 pathway, which separates the initial CO_2_ fixation and Calvin cycle processes over time (between day and night). The plant opens its stomata at night, allowing CO_2_ to diffuse into the leaves and be fixed as organic acids stored inside vacuoles until the next day, while the stomata would be shut to minimize photorespiration during the day. Meanwhile, the organic acids are transported from vacuoles and an enzyme releases the CO_2_ that enters into the Calvin cycle. The most important benefit of the CAM pathway is increased efficiency in the use of water in very hot and dry areas^22^.

Pineapple is an extremely economically and nutritionally valuable tropical fruit, and provides a suitable model to study obligate CAM photosynthesis in arid regions. Ming *et al*. has fully sequenced the pineapple genome with thorough annotations^23^. The availability of high quality genomic information and the increasing number of transcriptomic resources for pineapple make it an ideal system to globally identify the lncRNAs present in CAM photosynthesis. A previous study^23^ identified 38 putative genes associated with the carbon fixation module of CAM, including phosphoenolpyruvate carboxylase (PEPC) and phosphoenolpyruvate carboxylase kinase (PPCK), however, the regulatory elements involved in the CAM pathway remain largely unknown. Since miRNAs, lncRNAs, and ceRNAs are vital regulators of a multitude of biological processes, it is important to detect the regulatory affection for those core CAM enzymes.

In the present study, we conducted systematic identification and characterization of lncRNAs and identified a total of 2,888 putative leaf lncRNAs from the time-series of RNA-seq data of pineapple leaves. We validated our results by comparing genomic features of lncRNAs with these features of Arabidopsis, rice, or human lncRNAs, as well as to the pineapple protein-coding genes where appropriate, including exon numbers, exon length, transcript length, and tissue specific expression patterns. A co-expression network analysis indicated that many leaf lncRNAs are associated with photosynthesis genes. We also identified leaf lncRNAs that function as ceRNAs of two CAM pathway genes. We further found that lncRNAs have diurnal expression patterns in the pineapple leaf. Our genome-wide identification and further annotation of pineapple leaf lncRNAs will be beneficial for improving our knowledge of the molecular mechanisms that underlie the CAM pathway, as well as provide a perception of ceRNA-guided gene regulations in various biological processes in pineapple.

## Result

### Identification of putative leaf lncRNAs

**Figure 1:**
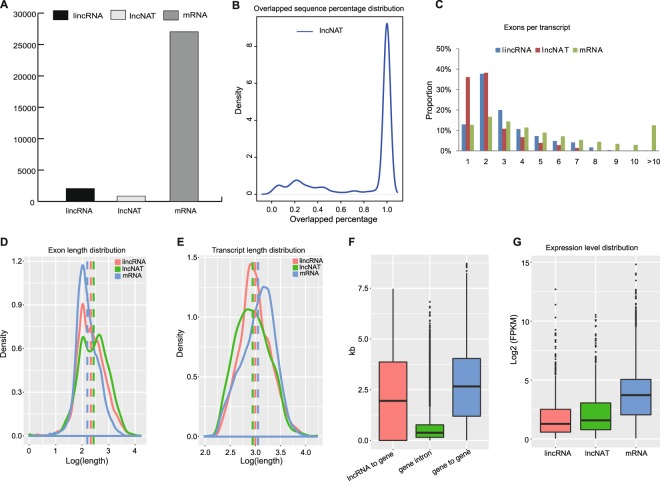
Properties of pineapple leaf lncRNAs. (**A**) The number of lincRNA, lncNAT and protein-coding genes. (**B**) The proportion of lncNAT sequences overlapped with sense genes in antisense direction. (**C**) Exon numbers for lincRNAs and lncNATs and protein-coding transcripts. (**D**) Exon length analysis for lincRNAs, lncNATs and protein-coding transcripts. (**E**) Transcript length analysis for lincRNAs, lncNATs and protein-coding transcripts. (**F**) Comparison distances of the mRNA–lincRNA intervals, mRNA–mRNA intervals, and length of mRNA introns. “lncRNA to gene” means the distance between lncRNA genes and their closest protein-coding genes, “gene intron” means the length of gene introns, and “gene to gene” represents distance between adjacent protein-coding genes. (**G**) The expression level of lincRNAs and lncNATs and protein coding genes.

To globally identify leaf lncRNAs related to the CAM pathway in pineapple, a modified computational method was used to mine putative lncRNAs using the leaf (green tip and whiter base) (Supplemental Fig. 1) RNA-seq datasets (the samples were collected at 2-h intervals through a 24-h period)^23,24^ (Supplemental Fig. 2). First, the clean reads (excluding low quality data) were aligned to the pineapple genomes (https://phytozome.jgi.doe.gov) using Tophat^25^. Second, we use Cufflinks to reconstruct the pineapple transcriptome from all of the RNA-seq datasets, which recovered a total of 117,031 transcripts in pineapple. Cuffmerge was then used to merge these assembled transcripts. The expression level of each transcript was estimated using Cuffdiff in each condition after the assembly of the whole transcriptome. The class codes of all transcripts were determined by Cuffcompare; only 7,420 (6.34%) of total transcripts with ‘u’, ‘x’, or ‘i’ code were selected to represent putative lncRNA candidates. Third, we retained 7,056 (6.03%) long (greater than 200 nucleotides) transcripts, according to the length criterion. Coding Potential Calculator (CPC)^26^ was used to perform the coding potential prediction for each transcript. Any transcripts with a CPC score >0 was excluded, resulting in 5,005 (4.28%) transcripts being retained. These transcripts were scanned in all three reading frames, and any transcript with known protein domain(s) in Pfam database was discarded^27^. At this phase, we were left with 4,878 (4.17%) lncRNA candidates. Finally, we kept only expressed transcripts with available strand information (multiple-exon lncRNAs with FPKM $\ge$0.5; single-exon lncRNAs with FPKM $\ge$2). Taken together, lncRNAs were defined as transcripts (1) with the length >200 nt; (2) CPC score <0; (3) do not have any Pfam domain; (4) have strand information; and (5) for multiple-exon transcripts FPKM ≥0.5, for single-exon transcripts FPKM ≥2 in at least one sample. With these criteria, we obtained 2,888 reliably expressed pineapple leaf lncRNAs (Supplemental Table 1), including 2,046 long intergenic noncoding RNAs (lincRNAs) and 842 long noncoding natural antisense transcripts (lncNATs) (Fig. 1A). Also, we considered that the filtered 1,990 novel transcribed loci without strandness or low expression as a set of low confidence lncRNAs, due to limited transcriptome data being available. Our newly identified leaf lncRNAs make it possible to further study their function, and provide a reliable reference to improve the gene annotation in pineapple.

### Pineapple leaf lncRNAs have distinct genomic features compared to protein-coding genes

The global genomic properties of lncRNAs have been studied in human and several model plant organisms (Arabidopsis and rice). However, such genome-wide information regarding lncRNA is still limited in pineapple. To examine main gene characteristics of lincRNAs, lncNATs and protein coding transcripts separately, we compared them mainly in the following aspects: exon numbers, exon length, transcript length, and tissue specific expression patterns. The results showed that lncNATs were overlapped with genes that were transcribed in antisense direction to the sense genes (Fig. 1B). Consistent with the results for humans^28^, most of lncRNAs were spliced (87% for lincRNAs, 63.9% for lncNATs), and show an obvious trend to have only two exons (37.73% for lincRNAs, 38.24% for lncNATs, compared with 16.66% of protein-coding genes) (Fig. 1C). On the contrary, only about half of rice and Arabidopsis lncRNAs were spliced^1,5,29^. The average number of exons of pineapple leaf lncRNAs is 2.80, while those of mRNAs is 5.53. Respectively, lincRNAs and lncNATs have 3.06 and 2.18 exons. Meanwhile, the median exon length of mRNA (137 nt) is shorter than that of lncRNAs (211 nt for lncRNAs, 198 nt for lincRNAs, and 282 nt for lncNATs) (Fig. 1D). Additionally, the median size of full-length lncRNA transcripts (920.5 nt for lncRNAs, 949.5 nt for lincRNAs and 850 nt for lncNATs) is longer than that in other species (Arabidopsis^1,29^, rice^5^, and human^28^), while the average length of all full-length mRNAs is longer (~1,227 nt) (Fig. 1E). The distance between leaf lncRNA genes and their closest protein-coding genes was shorter than the median distance between adjacent protein-coding genes (median 2,862 nt for lncRNA-gene intervals, compared with 5,320 nt for gene-gene intervals; Fig. 1F); while greater than the lengths of the introns in the protein-coding genes (Fig. 1F), indicating that these leaf lncRNAs are independent transcripts, rather than unannotated exons of these protein-coding genes. Furthermore, lncRNAs located closely to protein-coding genes modulate their expression by actively recruiting activators, repressors, epigenetic modifiers, or simply by transcription from the lncRNA locus.

FPKM values (fragments per kilobase of transcript sequence per million mapped reads) indicated that the lincRNAs and lncNATs have no obvious expression difference (median: 1.43 FPKM and 1.99 FPKM, respectively), while they were significantly lower than that of protein-coding genes (median: 12.20 FPKM, both P values < 1E-30, Kolmogorov–Smirnov test) (Fig. 1G). These features imply that lncRNAs and mRNAs may have several differences in their biogenesis, processing, stability, and spatial-temporal expression patterns.

### Co-expressed network reveals the association of leaf lncRNAs with photosynthesis genes

Co-expressed network construction is widely applied in large-scale lncRNA studies because it is useful for many purposes, such as candidate phenotype-based gene prioritization, functional gene annotation, and identification of regulatory gene partners^30^. We constructed the co-expression network using Pearson correlation coefficients (PCC) between pairwise leaf lncRNA and mRNA. In total, 18,436 interaction relationships (18,024 positive and 412 negative correlations) were identified between 700 lncRNA transcripts and 4,437 mRNAs in the pineapple genome (Supplemental Table 2). GO term enrichment results indicated that lncRNA co-expressed mRNAs were associated in microtubule-based and small molecule metabolic processes (Supplemental Table 3). Furthermore, the co-expressed genes were enriched in 9 KEGG pathways, several of which were related to photosynthesis, including glycolysis/gluconeogenesis, carbon fixation in photosynthetic organisms, and carbon metabolism (Supplemental Table 3). These findings indicate that leaf lncRNAs are associated with photosynthesis genes.

**Figure 2:**
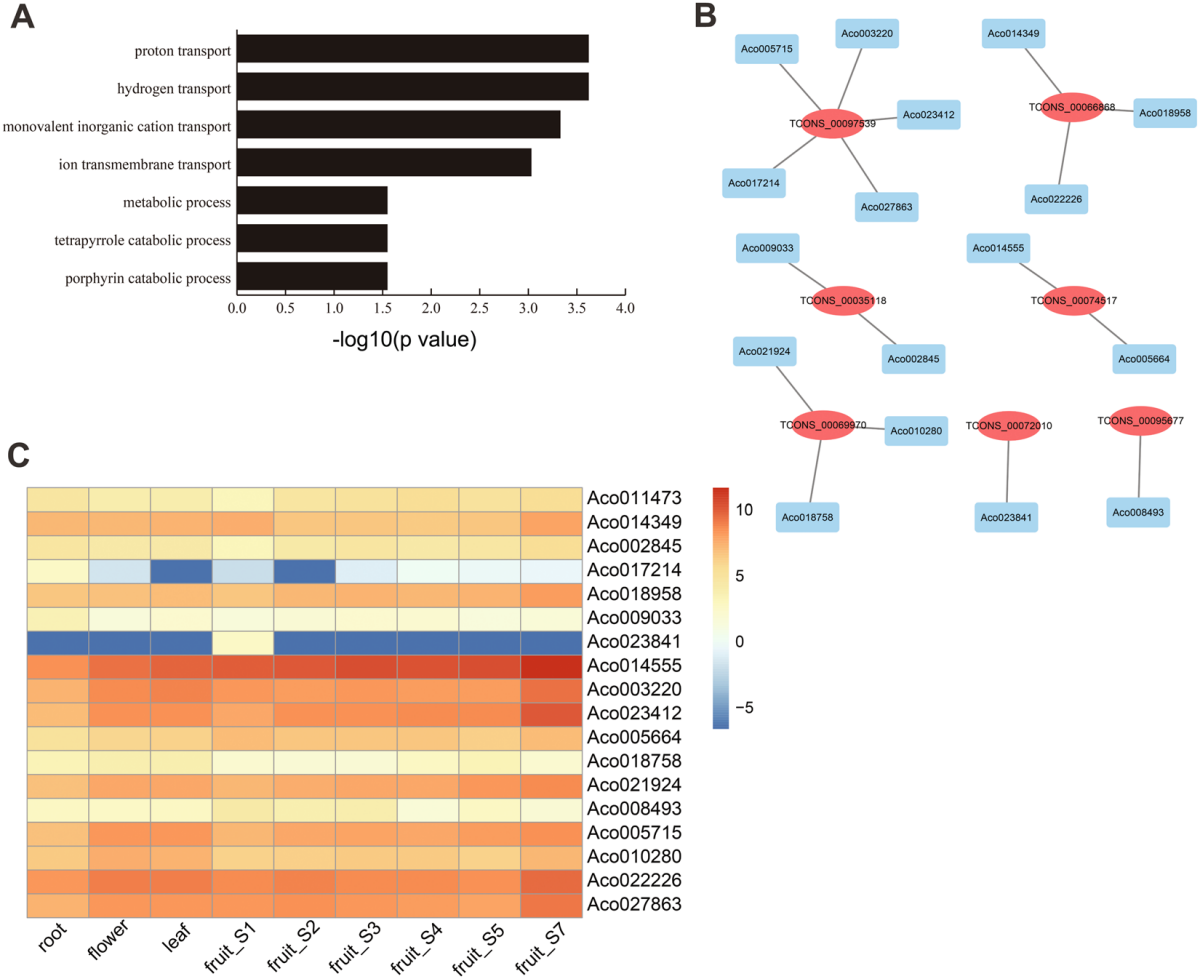
Analysis of lncRNA-ATP synthase family network. (**A**) GO enrichment for co-expressed genes. ATP synthase family genes were involved in the “proton and hydrogen transport” GO term. (**B**) The networks among lncRNAs and ATP synthase family genes. The red oval stands for lncRNAs and blue rectangle stands for ATP synthase family genes. (**C**) Heat maps of the ATP synthase family genes in different tissues. Red stands for high expression level and blue means low expression level.

To better understand the connection between biological nodes, differentially expressed genes (DEG), and leaf lncRNAs (DEL), samples were selected and mapped to the whole co-expression network. As shown in Supplemental Fig. 3, the DEG-DEL co-expression network consisted of 2,160 edges between 1,450 network nodes (1,406 genes and 44 lncRNAs). The results showed that most of the gene-lncRNA pairs were positively correlated (2,151 positive interactions and 9 negative interactions between pairs within the network) (Supplemental Table 4). Moreover, one mRNA may correlate with 1 to 8 lncRNAs, while one lncRNA may correlate with 1 to 406 mRNAs (Supplemental Fig. 3). Functional analysis showed that the co-expressed genes were enriched in proton transport, hydrogen transport, and monovalent inorganic cation transport (Fig. 2A). Proton transport and hydrogen transport GO terms were highly represented among all GO terms, which contain 18 genes (Supplemental Table 5). Interestingly, each of these 18 genes is a member of the ATP synthase or V-ATPase gene family. Their expression levels were consistently high in different tissues, except for Aco017214 and Aco023841 (Fig. 2B). Using co-expression analysis and network construction in Cytoscape 3.5.0, we found that these 18 ATP synthase or V-ATPase genes might be regulated by 7 of the identified DELs (Fig. 2C). For example, TCONS_00097539 was identified as regulator of Aco005715, Aco003220, Aco023412, Aco027863, and Aco017214, while TCONS_00066868 could target Aco014349, Aco018958, and Aco022226.

### LincRNAs show highly tissue-specific expression pattern

**Figure 3:**
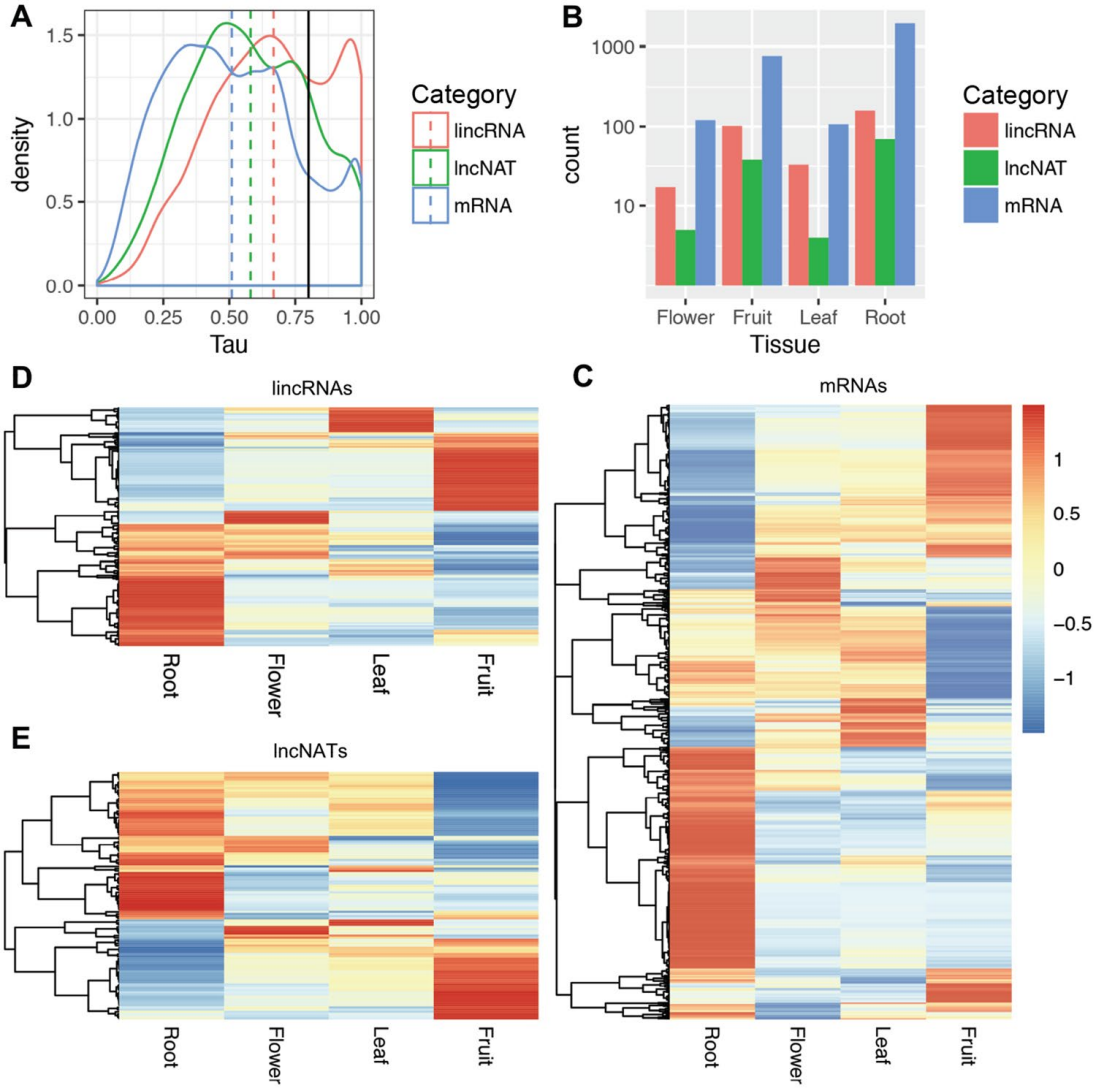
Tissue specific lincRNA expression analysis. (**A**) LincRNAs tend to be far more tissue-specific than mRNAs by measuring tau score. Dashed line stands for the median tau score. The higher the tau score, the higher the expression level. The black solid line stands for tau score 0.8 and tau score larger than 0.8 was considered as tissue-specific genes. (**B**) Number of total lincRNA, lncNAT and mRNA genes expressed in each tissue (fruits from different time points were combined). (**C**–**E**) Heatmaps of tissue specific expressed mRNAs, lincRNAs and lncNATs. Red color means high and blue means low expression level.

**Figure 4:**
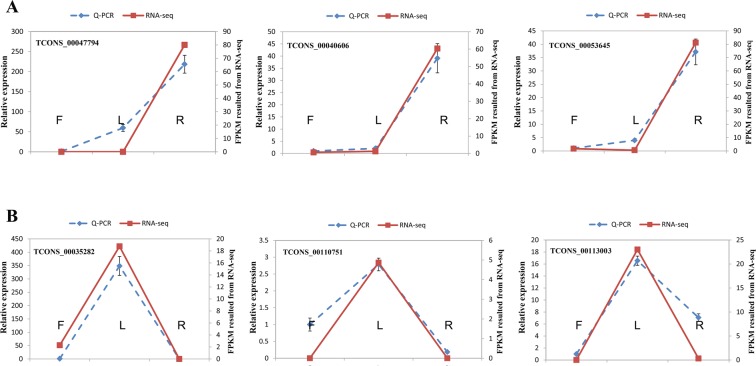
Validation of six random selected root and leaf tissue specific expressed lncRNAs by RT-qPCR. The left y axis represents for the relative expression from RT-qPCR result and right y axis stands for the FPKM value from RNA-seq result. Data are the mean ± SEM. Blue dash line represents for Q-PCR and red solid line represents for RNA-seq result. The letter F, L and R means flower, leaf and root tissues. (**A**) Results for three root specific expressed lncRNAs. (**B**) Validation for three leaf specific expressed lncRNAs.

Many lncRNAs exert their functions in a tissue-specific manner to regulate biological processes^31^. To characterize and compare the expression pattern of pineapple leaf lincRNAs, lncNATs, and mRNAs in different tissues, we used RNA-seq data sets from flower, leaf, root, and fruit (average expression level of six development stages)^23^. Here, we use Tau score to indicate the tissue specificity of gene expression, which range from 0 (no specificity) to 1 (high specificity)^32^. Firstly, we filtered out the low expression lncRNAs and protein-coding genes (FPKM <1 in all tissues). 13.5% mRNAs (2955/20261) and 26.9% lncRNAs (310/950 for lincRNA and 116/632 for lncNAT) with Tau score larger than 0.8 were considered as tissue-specific genes (Supplemental Table 6). The results revealed that lincRNAs have a significant tendency to be more tissue-specifically expressed than mRNAs (Kolmogorov-Smirnov test, *P* = 0.0077), while lncNATs showed no obvious tissue-specific expression pattern compared to that of mRNAs (Fig. 3A). A similar trend was observed using entropy (*Hg* score) as a tissue specificity measurement (Supplemental Fig. 4)^33^. The highly tissue-specific expression pattern of lincRNAs may provide an opportunity to classify lincRNAs according to their expression patterns. Only 22 lincRNAs and 21 lncNATs were detected in all the samples. Interestingly, a considerable amount of pineapple lncRNAs are specifically expressed at a single development stage. 150 uniquely expressed lncRNAs are detected in root (105 lincRNA, 45 lncNAT), 19 in flower (15 lincRNA, 4 lncNAT), 27 in leaf (25 lincRNA, 2 lncNAT), and 109 in fruit (83 lincRNA, 26 lncNAT). We found that root contained the largest number of tissue specific mRNAs (1,961), followed by fruit (767), flower (120), and leaf (107) (Fig. 3B). The expression profiles of tissue-specific genes in the 4 tissues are shown in Fig. 3C. Tissue-specific analysis was also performed for lncRNAs. The results showed that root also contained the largest number of tissue-specific expressed lncRNAs (158 lincRNA, 69 lncNAT), followed by fruit (102 lincRNA, 38 lncNAT), flower (17 lincRNA, 5 lncNAT), and leaf (33 lincRNA, 4 lncNAT) (Fig. 3B). The expression levels of these lincRNAs and lncNATs in 4 tissues are shown by heatmap (Fig. 3D,E). To validate the expression patterns of the lncRNAs, we randomly selected 6 tissue-specific expressed lncRNAs in root (TCONS_00047794, TCONS_00040606, TCONS_00053645) (Fig. 4A) and leaf (TCONS_00035282, TCONS_0011075, TCONS_00113003) (Fig. 4B), and confirmed their expression level using real-time quantitative PCR (qRT-PCR). The experimental results were consistent with our RNA-seq results, suggesting that the lncRNAs expression patterns based on RNA-seq data are reliable.

### Identification of leaf lncRNAs that function as ceRNAs of two CAM pathway genes

Previous studies shown that lncRNAs can act as ceRNAs by binding to and isolating specific miRNAs in a type of target mimicry to prevent the target of mRNAs from repression in both plants and animals^34,35^. We predicted lncRNAs that might function as ceRNAs using the algorithm developed by Yuan *et al*.^36^. We found 73,486 potentially ceRNA target-target pairs, which are associated with 125 lincRNAs and 47 lncNATs, and identified 636 target-mimic pairs, which are associated in 163 lincRNAs and 76 lncNATs in pineapple (Supplemental Table 7).

**Figure 5:**
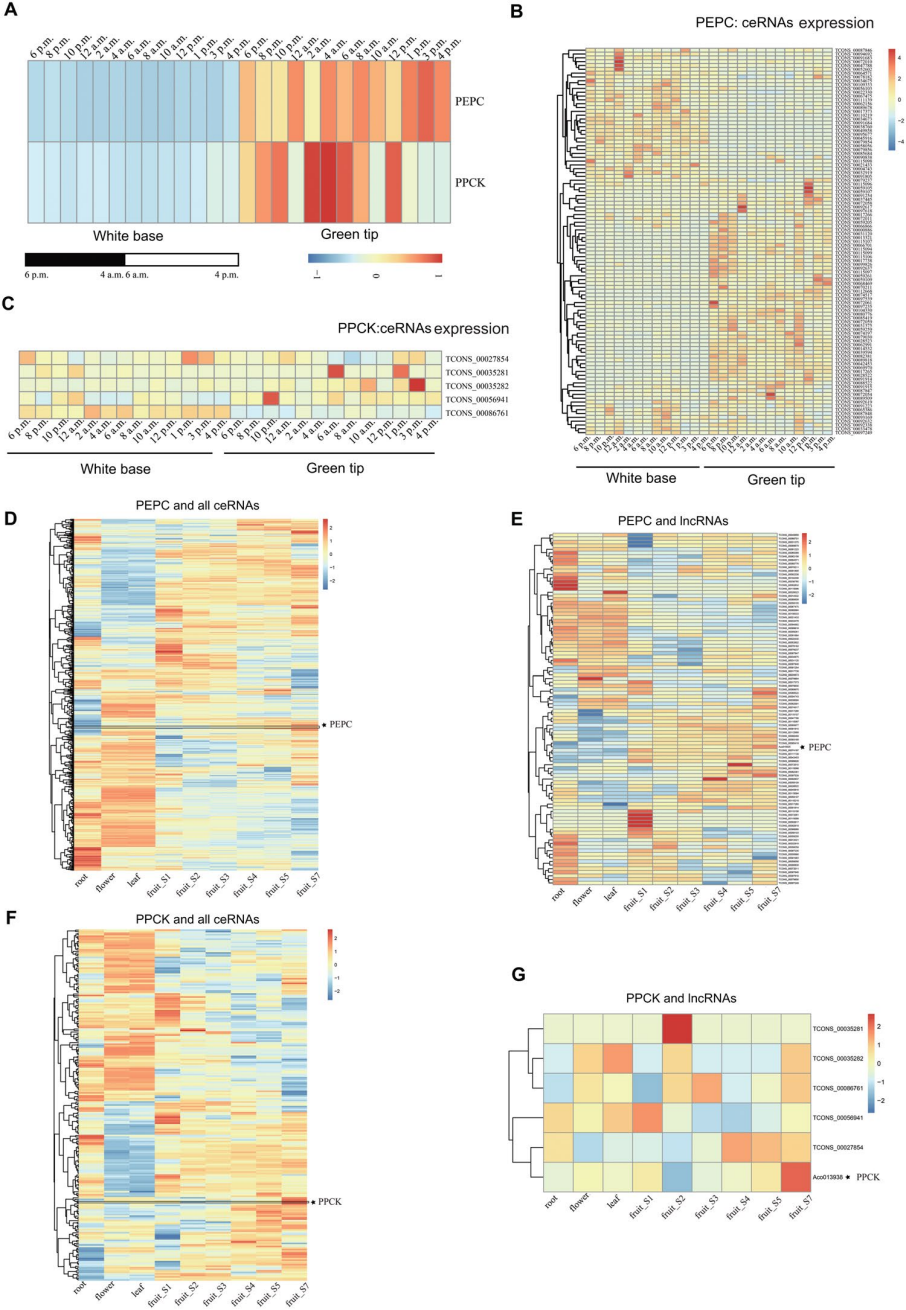
Identification of ceRNAs for two important CAM pathway enzymes (PEPC and PPCK). (**A**) Heatmap for PEPC and PPCK genes expression in white base and green tip of pineapple leaves at different time points. (**B**) Expression pattern for putative ceRNAs (only the lncRNAs) of PEPC at different time points. (**C**) Heatmap for putative ceRNAs (only the lncRNAs) of PPCK. (**D**) The expression patterns of all PEPC ceRNAs in root, flower, leaf and fruits. (**E**) The expression patterns of putative PEPC ceRNAs (only the lncRNAs). (**F**) The expression patterns of all PPCK ceRNAs in root, flower, leaf and fruits. (**G**) The expression patterns of putative PPCK ceRNAs (only the lncRNAs). The locations of PEPC and PPCK in the heatmap were indicated by star shape.

**Figure 6:**
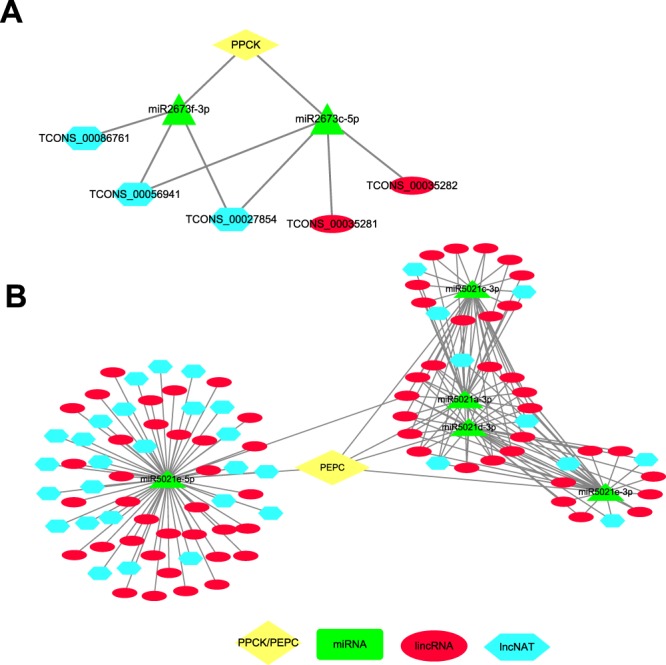
The construction of ceRNA networks. (**A**) The network for PPCK and their ceRNA pairs. Two lincRNAs and three lncNATs competed two miRNAs to regulate PPCK expression. (**B**) The network for PEPC and their ceRNA pairs. 101 lncRNAs competed five miRNAs to regulate PEPC expression. The yellow diamond means PPCK/PEPC. Green triangle represents miRNAs, red oval is for lincRNAs and light blue hexagon is for lncNATs.

A previous study identified 38 putative genes that are associated in the carbon fixation module of CAM, such as the key carbonic anhydrase (*CA*), phosphoenolpyruvate carboxylase (*PEPC*), and phosphoenolpyruvate carboxylase kinase (*PPCK*)^23^. To investigate the diel expression patterns of CAM pathway genes, we found that nine genes had a diurnal expression pattern in the green leaf tissue while low or no expression in the white leaf tissue^23^, for example *PPCK* and *PEPC* genes (Fig. 5A). In this study, we predicted 101 lncRNAs functioning as ceRNAs of *PEPC* gene (Fig. 5B) and 5 lncRNAs functioning as ceRNAs of *PPCK* gene (Fig. 5C). The expression of five lncRNAs (two lincRNAs and three lncNATs) acted as putative ceRNAs, which could compete for binding to two miRNAs (miR2673f-3p and miR2673c-5p), and could also result in an up-regulation of PPCK mRNA levels that effect the CAM pathway (Fig. 6A). As shown in Fig. 6B, 101 lncRNAs competed for 5 miRNAs (miR5021e-5p, miR5021c-3p, miR5021a-3p, miR5021d-3p and miR5021e-3p) to release PEPC mRNA from repression. This ceRNA network, involved in PPCK/PEPC, lncRNAs and miRNAs, indicated that lncRNAs might provide another level of regulation for the CAM pathway. Additionally, the ceRNAs in *PPCK* and *PEPC* genes at two hour intervals over a 24-hour period exhibited a diurnal expression pattern (Fig. 5B,C), suggesting that lncRNAs in CAM carbon fixation are involved with the circadian clock. We further analyzed the expression pattern of all these *PPCK* and *PEPC* ceRNAs in different tissues, including root, flower, leaf, and six fruit development stages, and found that most of the ceRNAs also exhibited tissue specific expression patterns (Fig. 5D–G).

### Diurnal expression pattern of pineapple leaf lncRNAs

**Figure 7:**
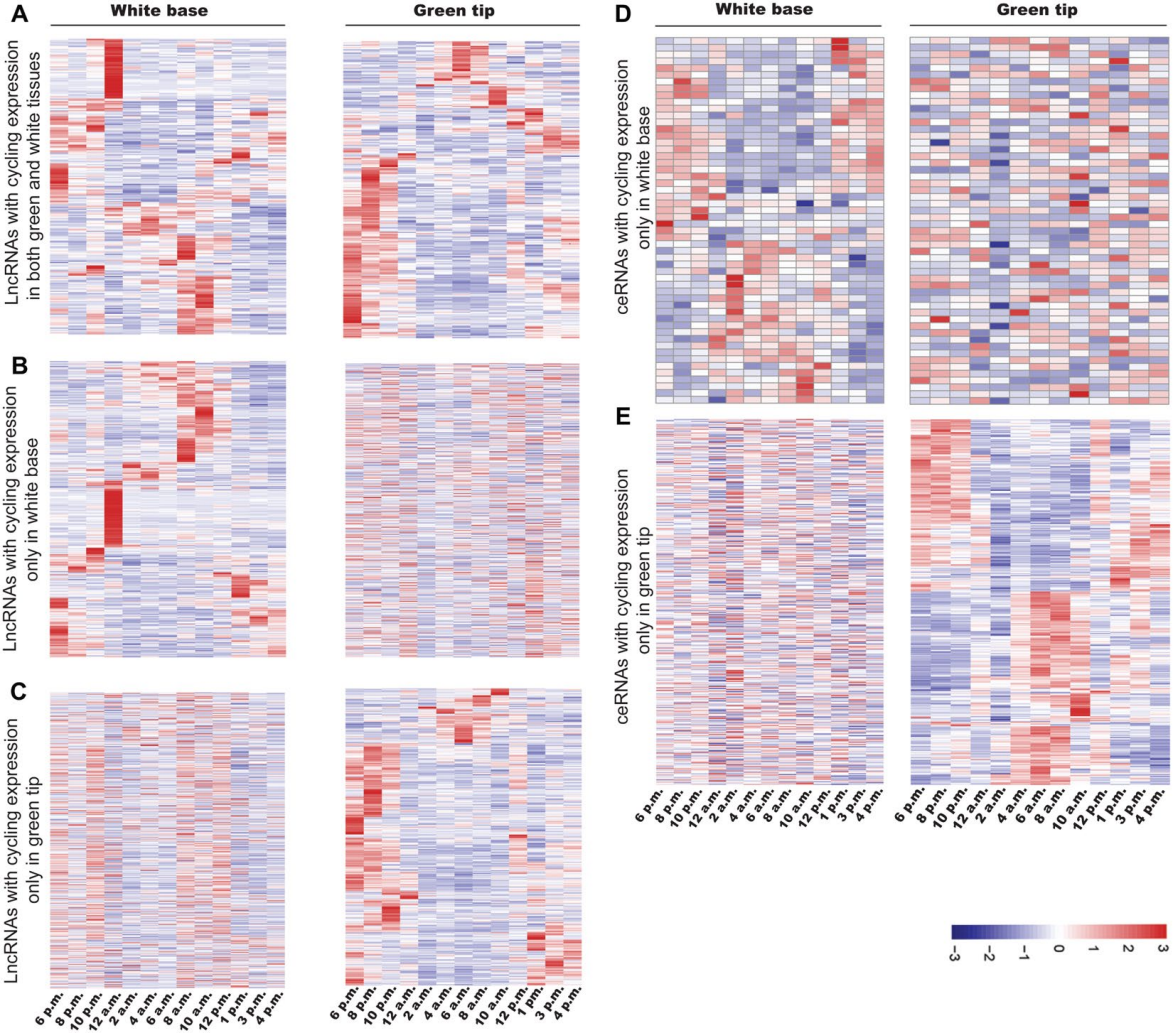
Diurnal expression profiles of cycling lncRNAs in pineapple leaf green tip and white base tissues. Red represents the highest expression, blue represents the lowest expression, and white represents an intermediate expression. (**A**) LncRNAs with cycling expression in both white base and green tip. (**B**) LncRNAs with cycling expression only in white base. (**C**) LncRNAs with cycling expression only in green tip. (**D**) Diurnal expression profiles of cycling ceRNAs with a diel peak expression only in white base (**E**) ceRNAs with cycling expression pattern only in green tip. X axis stands for different time points in white base/green tip. Y axis means for cycling expressed lncRNAs or ceRNAs.

Most clock component and clock-regulated genes display diurnal expression patterns, which generate the circadian rhythms in plant. We used the Haystack algorithm^37^ to detect lncRNAs and ceRNAs whose diel expression patterns fit a predefined model of cycling genes. We used the tailored models to adapt our collection time points (two hour intervals over a 24-hour period) according to the models defined by Endo *et al*.^38,39^. We empirically defined cycling lncRNAs and ceRNAs as those with a strong correlation (r > 0.7) to a predefined model of cycling genes (fold change >2, P value > 0.05, and amplitude >10). In accordance with this criterion, 48% of lncRNAs (1,390 out of 2,888) were shown to be cycling in either one or both green tip and white base leaf tissues, including 257 (9%) cycling in both tissues (Fig. 7A, Supplemental Table 8), 552 (19%) cycling in the white leaf base only (Fig. 7B, Supplemental Table 8) and 581 (20%) cycling in the green tissue only (Fig. 7C, Supplemental Table 8). Additionally, we identified 54 ceRNAs cycling only in white leaf base and 404 ceRNAs cycling only in green tissue (Supplemental Table 9). Diurnal expression profiles of cycling ceRNAs with a diel peak expression in white base and green tip are shown in Fig. 7D,E. Based on our results, it is reasonable to assume that circadian expression patterns of leaf lncRNAs may also be key regulators of diurnal oscillations of physiological and metabolic processes, including photosynthetic enzyme activity in pineapple.

## Discussion

Pineapple is an extremely nutritionally and economically valuable tropical fruit, and a suitable model for studying obligate CAM photosynthesis evolved in plants grown in arid regions. In the recently published pineapple genome, about 27,000 protein coding genes were reported^23^, providing material support for the characterization of CAM pathway genes. The time series deep sequencing data of pineapple leaf tissues reported in the pineapple genome study^23^ offers an opportunity for the identification and characterization of pineapple leaf lncRNAs involved in CAM photosynthesis. Studies in human^40,41^ and other animal model organisms^42,43^ have demonstrated the important roles of lncRNA in various biological processes. While a recent study identified pineapple lncRNAs in leaf and stem apex tissues^44^, our knowledge still remains limited regarding the spatial-temporal transcriptional dynamics of lncRNAs in pineapple leaf and the role of lncRNAs in the CAM pathway. In this study, by using a computational pipeline that we developed to analyze RNA-seq data, we identified 2,888 leaf lncRNAs, including 2,046 lincRNAs and 842 lncNATs in the pineapple green tip and white base leaf tissues of a time-series at 24-hour time periods with two hour intervals. The amount of pineapple leaf lncRNAs identified in this study is comparable to that in rice (2,224 lncRNAs)^5^ and chickpea (2,248 lincRNA)^45^. Wang *et al*. identified more than 12,000 lncRNA transcripts in both pineapple leaf and stem apex tissues by Pacbio ISO-seq technology^44^, among which about 3,000 lncRNAs cannot be detected by Illumina short-reads sequencing, which was used for the pineapple time-series of leaf tissue RNA-seq analysis^23^. These differences could be due to varying tissues used for studies and the different techniques applied.

Nevertheless, the identified pineapple leaf lncRNAs in this study share most common features with other species including Arabidopsis^3^, rice^5^, maize^6^ and soybean^12^. LncRNAs have shorter length and lower expression levels than protein-coding transcripts. Pathway enrichment analysis of the *cis*-regulated target genes of lncRNAs and GO term enrichment analysis of lncRNA co-expressed mRNAs showed that the lncRNAs identified in pineapple leaf tissues are preferentially associated with photosynthesis, suggesting the highly specified function of lncRNAs in leaves. Studies across species showed that lncRNAs exert their functions in a tissue-specific manner to regulate biological processes^46^. In this study, we further analyzed the RNA-seq data sets from flower, leaf, root, and fruit tissues and found that the expression pattern of pineapple lincRNAs exhibit more tissue specific manner than mRNAs, similar to the finding in other species^28,41,43,47,48^. Among the four analyzed pineapple tissues, we found that root contained the largest number of tissue-specific expressed lncRNAs, followed by fruit, flower, and lastly leaf. In soybean, the highest number of tissue-specific expressed lncRNAs is detected in flower^12^, and the largest number of lincRNAs is accumulated in the shoot apical meristem in chickpea^45^. The high tissue-specific lncRNA expression pattern indicates their highly specialized, possible regulatory functions. It also implies the potential use of lncRNAs as tissue type and physiological state markers.

The CAM metabolic pathway is found mainly in plants that grow in arid climates^49^. It allows the plant to open its stomata to collect and store CO_2_ during the night and release it the next day for photosynthesis, thus improving water-use efficiency and drought resistance through keeping its stomata closed during the day^50^. Recent studies reveal that the circadian rhythm of CAM is regulated by the circadian clock^51^. Here, we found that lncRNAs in pineapple leaf tissues are preferentially associated with photosynthesis genes, and around half (48%) of the lncRNAs show diurnal expression patterns, similar to the clock-regulated genes, which exhibit diurnal expression patterns and are responsible for generating the circadian rhythms in the plant^52^. It is reasonable to speculate that this circadian expression of leaf lncRNAs may also be involved in the regulation of diurnal oscillations of physiological and metabolic processes, including photosynthetic enzyme activity in pineapple^23^. There are 38 putative genes involved in the carbon fixation module of CAM in pineapple, including *PEPC* and *PPCK*, which showed diel expression patterns. To investigate the role of lncRNA in the carbon fixation module and the regulation of these two genes, we predicted that lncRNAs function as ceRNAs. We found 101 and 5 leaf lncRNAs that could act as ceRNAs of *PEPC* and *PPCK*, respectively, likely by binding to and sequestering specific miRNAs to protect these genes from repression^36^. The ceRNAs of *PPCK* and *PEPC* genes also exhibited a diurnal expression pattern, suggesting that the involvement of leaf lncRNAs in carbon fixation in CAM is associated with the circadian clock.

Our findings demonstrate that leaf lncRNAs play an important role in pineapple photosynthesis and development network. Our study provides evidence of the role of lncRNAs in different pineapple tissues and provides a new perspective on the regulatory mechanisms in which they are involved. The identification and characterization of the lncRNAs would strongly benefit the annotation of the pineapple reference genome and lead to a better understanding of the biological basis of regulatory interactions amongst mRNAs, miRNAs, and lncRNAs.

## Materials and Methods

### Data sources

Pineapple genome assembly *Ananas comosus* v3 was used throughout this study and was downloaded from Phytozome v12 (https://phytozome.jgi.doe.gov). All the RNA-seq datasets used in this study were obtained from a previous publication^23^. The transcriptome data contains temporal gene expression profiling of green leaf tip and white leaf base at 13 time points (26 samples, three biological replicates per sample), including 6 P.M., 8 P.M., 10 P.M., midnight, 2 A.M., 4 A.M., 6 A.M., 8 A.M., midday, 1 P.M., 3 P.M. and 4 P.M.

### Co-expression analysis

We used the expression levels of the identified putative lncRNAs and known protein-coding genes from 26 time points series samples to analyze their co-expression. We calculated Pearson’s correlation coefficients between the expression levels of 2,888 lncRNAs and 18,921 protein-coding genes with custom scripts (r > 0.95 or r < −0.95). Then, we performed a functional enrichment analysis of the candidate lncRNA target genes using BINGO and ClueGO software. A P-value < 0.05 was considered significant.

### Confirmation of lncRNA expression by qRT-PCR experiments

Total RNAs were extracted from pineapple leaf, root, and flower tissues and then transcribed reversely using the PrimeScriptTM RT reagent kit (Takara, Otsu, Shiga, Japan). Real-time PCR was conducted using SYBR Premix Ex Taq™ (Takara). Actin2 was used as a reference gene. Real-time PCR was carried out according to the manufacturer’s instructions (Takara). The specificity of PCR product was reflected by the single peak melting curves. The comparative Ct method was applied for the quantification of lncRNA expression. These assays were conducted for three biological replicates, and the results are shown as the mean ± standard deviations.

### Identification of tissue specific mRNA and leaf lncRNAs

Another set of RNA-seq data (including flower, leaf, root, and fruit) in pineapple was downloaded and analyzed as previously described. Expression level of both mRNAs and lncRNAs was quantified by Cuffllinks, with multiple expression values in fruit averaged. Transcripts with low expression (FPKM < 1 in all tissues) were discarded. Tau score (${\rm{\tau }}$) was used to measure tissue specificity of gene expression as described by Yanai *et al*.^33^:

$${\rm{\tau }}=\frac{{\sum }_{i=1}^{N}(1-\widehat{{x}_{\iota }})}{N-1};\,\widehat{{x}_{\iota }}=\frac{{x}_{i}}{\mathop{{\rm{\max }}}\limits_{1\le i\le N}({x}_{i})}$$

where *N* is the number of tissues and $\widehat{{x}_{\iota }}$ is the expression profile component normalized by the maximal component value. The values of tau vary from 0 to 1, where 0 means ubiquitous expressed transcripts and 1 specific transcript.

### Prediction of leaf lncRNAs that might function as ceRNAs

In accordance with Yuan’s method^36^, we performed three steps to predict lncRNAs to be a putative ceRNA. Firstly, we used TargetFinder to identify all pineapple miRNAs target transcripts. Secondly, we detected all miRNAs that could bind our lncRNAs through the results of TargetFinder and Tapir. TargetFinder was used to predict target transcripts perfectly bound by miRNAs, while Tapir was used to identify putative target mimics (imperfect binding). If two transcripts were bound by the same miRNA(s), these two transcripts represented a ceRNA pair. Target-target pairs mean miRNAs could perfectly bind to the ceRNAs, while target-mimic pairs represent imperfect binding.

## Supplementary information

Supplementary information

Supplementary Dataset 9

## References

[1] Liu J (2012). Genome-wide analysis uncovers regulation of long intergenic noncoding RNAs in Arabidopsis. Plant Cell.

[2] Rymarquis LA, Kastenmayer JP, Huttenhofer AG, Green PJ (2008). Diamonds in the rough: mRNA-like non-coding RNAs. Trends Plant Sci.

[3] Song D (2009). Computational prediction of novel non-coding RNAs in Arabidopsis thaliana. BMC Bioinformatics.

[4] Di C (2014). Characterization of stress-responsive lncRNAs in Arabidopsis thaliana by integrating expression, epigenetic and structural features. Plant J.

[5] Zhang YC (2014). Genome-wide screening and functional analysis identify a large number of long noncoding RNAs involved in the sexual reproduction of rice. Genome Biol.

[6] Boerner S, McGinnis KM (2012). Computational identification and functional predictions of long noncoding RNA in Zea mays. PLoS One.

[7] Wen J, Parker BJ, Weiller GF (2007). In Silico identification and characterization of mRNA-like noncoding transcripts in Medicago truncatula. In Silico Biol.

[8] Xin M (2011). Identification and characterization of wheat long non-protein coding RNAs responsive to powdery mildew infection and heat stress by using microarray analysis and SBS sequencing. BMC Plant Biol.

[9] Wang L (2013). Deep RNA-Seq uncovers the peach transcriptome landscape. Plant Mol Biol.

[10] Chen J, Quan M, Zhang D (2015). Genome-wide identification of novel long non-coding RNAs in Populus tomentosa tension wood, opposite wood and normal wood xylem by RNA-seq. Planta.

[11] Shuai P (2014). Genome-wide identification and functional prediction of novel and drought-responsive lincRNAs in Populus trichocarpa. J Exp Bot.

[12] Golicz AA, Singh MB, Bhalla PL (2018). The Long Intergenic Noncoding RNA (LincRNA) Landscape of the Soybean Genome. Plant Physiol.

[13] Yu, X. *et al*. Global analysis of cis-natural antisense transcripts and their heat-responsive nat-siRNAs in Brassica rapa. *BMC Plant Biol*. **13**, 10.1186/1471-2229-13-208 (2013). 10.1186/1471-2229-13-208 PMC4029752 24320882

[14] Wutz A, Gribnau JX (2007). Inactivation Xplained. Curr Opin Genet Dev.

[15] Bardou F (2014). Long noncoding RNA modulates alternative splicing regulators in Arabidopsis. Dev Cell.

[16] Yuan J (2016). Systematic characterization of novel lncRNAs responding to phosphate starvation in Arabidopsis thaliana. BMC genomics.

[17] Wierzbicki AT (2012). The role of long non-coding RNA in transcriptional gene silencing. Curr Opin Plant Biol.

[18] Salmena L, Poliseno L, Tay Y, Kats L, Pandolfi PP (2011). A ceRNA hypothesis: the Rosetta Stone of a hidden RNA language?. Cell.

[19] Militello G (2017). Screening and validation of lncRNAs and circRNAs as miRNA sponges. Brief Bioinform.

[20] Herrera A (2009). Crassulacean acid metabolism and fitness under water deficit stress: if not for carbon gain, what is facultative CAM good for?. Ann. Bot..

[21] Herrera A (2008). Crassulacean acid metabolism and fitness under water deficit stress: if not for carbon gain, what is facultative CAM good for?. Ann. Bot..

[22] Vandegrift, D. A. *Expanding the Plant Palette for Green Roofs*. (Michigan State University, 2018).

[23] Ming R (2015). The pineapple genome and the evolution of CAM photosynthesis. Nat Genet.

[24] Wai CM (2017). Temporal and spatial transcriptomic and micro RNA dynamics of CAM photosynthesis in pineapple. The Plant Journal.

[25] Trapnell C (2012). Differential gene and transcript expression analysis of RNA-seq experiments with TopHat and Cufflinks. Nat Protoc.

[26] Kong L (2007). CPC: assess the protein-coding potential of transcripts using sequence features and support vector machine. Nucleic Acids Res..

[27] Punta M (2012). The Pfam protein families database. Nucleic Acids Res..

[28] Derrien T (2012). The GENCODE v7 catalog of human long noncoding RNAs: analysis of their gene structure, evolution, and expression. Genome Res..

[29] Wang H (2014). Genome-wide identification of long noncoding natural antisense transcripts and their responses to light in Arabidopsis. Genome Res..

[30] van Dam, S., Vosa, U., van der Graaf, A., Franke, L. & de Magalhaes, J. P. Gene co-expression analysis for functional classification and gene-disease predictions. *Brief Bioinform*, 10.1093/bib/bbw139 (2017). 10.1093/bib/bbw139 PMC6054162 28077403

[31] Joung J (2017). Genome-scale activation screen identifies a lncRNA locus regulating a gene neighbourhood. Nature.

[32] Kryuchkova-Mostacci N, Robinson-Rechavi M (2016). Tissue-Specificity of Gene Expression Diverges Slowly between Orthologs, and Rapidly between Paralogs. PLoS Comput Biol.

[33] Kryuchkova-Mostacci N, Robinson-Rechavi M (2017). A benchmark of gene expression tissue-specificity metrics. Brief Bioinform.

[34] Franco-Zorrilla JM (2007). Target mimicry provides a new mechanism for regulation of microRNA activity. Nat Genet.

[35] Wang J (2010). CREB up-regulates long non-coding RNA, HULC expression through interaction with microRNA-372 in liver cancer. Nucleic Acids Res..

[36] Yuan C (2017). PceRBase: a database of plant competing endogenous RNA. Nucleic Acids Res..

[37] Mockler TC (2007). The DIURNAL project: DIURNAL and circadian expression profiling, model-based pattern matching, and promoter analysis. Cold Spring Harb Symp Quant Biol.

[38] Endo M, Shimizu H, Nohales MA, Araki T, Kay SA (2014). Tissue-specific clocks in Arabidopsis show asymmetric coupling. Nature.

[39] Sharma A, Wai CM, Ming R, Yu Q (2017). Diurnal Cycling Transcription Factors of Pineapple Revealed by Genome-Wide Annotation and Global Transcriptomic Analysis. Genome Biol Evol.

[40] Iyer MK (2015). The landscape of long noncoding RNAs in the human transcriptome. Nat Genet.

[41] Cabili MN (2011). Integrative annotation of human large intergenic noncoding RNAs reveals global properties and specific subclasses. Genes Dev..

[42] Lv J (2015). Identification of 4438 novel lincRNAs involved in mouse pre-implantation embryonic development. Molecular genetics and genomics: MGG.

[43] Pauli A (2012). Systematic identification of long noncoding RNAs expressed during zebrafish embryogenesis. Genome Res..

[44] Wang J (2017). Integrated DNA methylome and transcriptome analysis reveals the ethylene-induced flowering pathway genes in pineapple. Sci Rep.

[45] Khemka N, Singh VK, Garg R, Jain M (2016). Genome-wide analysis of long intergenic non-coding RNAs in chickpea and their potential role in flower development. Sci Rep.

[46] Till, P., Mach, R. L. & Mach-Aigner, A. R. A current view on long noncoding RNAs in yeast and filamentous fungi. *Appl Microbiol Biotechnol*, 10.1007/s00253-018-9187-y (2018). 10.1007/s00253-018-9187-y PMC6097775 29974182

[47] Nam JW, Bartel DP (2012). Long noncoding RNAs in C. elegans. Genome Res..

[48] Zhong L (2018). Long non-coding RNAs involved in the regulatory network during porcine pre-implantation embryonic development and iPSC induction. Sci Rep.

[49] Yang X (2015). A roadmap for research on crassulacean acid metabolism (CAM) to enhance sustainable food and bioenergy production in a hotter, drier world. New Phytol.

[50] Males J, Griffiths H (2017). Stomatal Biology of CAM Plants. Plant Physiol.

[51] Boxall SF, Dever LV, Knerova J, Gould PD, Hartwell J (2017). Phosphorylation of Phosphoenolpyruvate Carboxylase Is Essential for Maximal and Sustained Dark CO_2_ Fixation and Core Circadian Clock Operation in the Obligate Crassulacean Acid Metabolism Species Kalanchoe fedtschenkoi. Plant Cell.

[52] Dalchau N (2011). The circadian oscillator gene GIGANTEA mediates a long-term response of the Arabidopsis thaliana circadian clock to sucrose. Proc. Natl. Acad. Sci. USA.

