# Supplementary material for: Identification and characterization of pineapple leaf lncRNAs in crassulacean acid metabolism (CAM) photosynthesis pathway
Source: Sci Rep. 2019 Apr 30;9:6658. doi: 10.1038/s41598-019-43088-8 (PMC6491598; doi:10.1038/s41598-019-43088-8)
Supplement: Supplementary file 1 — Supplementary information [file 41598_2019_43088_MOESM1_ESM.pdf]

Supplementary Information

Identification and characterization of pineapple leaf lncRNAs in crassulacean acid metabolism (CAM) photosynthesis pathway

Youhuang Bai[1,5], Xiaozhuan Dai[1,5], Yi Li[1,5], Lulu Wang[1,3], Weimin Li[1,3], Yanhui Liu[1], Yan Cheng[1,4], Yuan Qin[2*]

[1]College of life science, Fujian Agriculture and Forestry University, Fuzhou 350002, China

[2]State Key Laboratory for Conservation and Utilization of Subtropical Agro-Bioresources, Guangxi Key Lab of Sugarcane Biology, College of Agriculture, Guangxi University, Nanning 530004, Guangxi, China

[3]College of Resources and Environment, Fujian Agriculture and Forestry University, Fuzhou 350002, China

[4]College of Plant Protection, Fujian Agriculture and Forestry University, Fuzhou 350002, China

[5]Equally contributed to this work

*Correspondence: Yuan Qin (yuanqin@fafu.edu.cn)

Supplemental Figure 1. The pineapple leaf that used to collect RNA-seq samples is shown. The white base and green tip tissue used in the RNA-seq experiment are label on the bottom.

Supplemental Figure 2. An integrative computational pipeline for the systematic identification of lncRNAs in pineapple. CPC, Coding Potential Calculator; ME, multiple exon; SE, single exon.

Supplemental Figure 3. The whole co-expression network profiles among 1406 differentially expressed genes and 44 differentially expressed lncRNAs (DELs). The red nodes stand for lncRNAs and blue boxes represent protein coding genes. The positive connections are showed by black line and negative connections are showed by purple line.

Supplemental Figure 4. The scatter plot of tissue specificity measured by two methods: Tau score and Hg score.

Supplemental Table 1. The information of predicated long noncoding RNAs.

Supplemental Table 2. Co-expression analysis between pairwise lncRNAs and mRNAs.

Supplemental Table 3. GO enrichment and KEGG analysis for *trans*-regulatory target genes.

Supplemental Table 4. The co-expression network analysis for differentially expressed genes and differentially expressed lncRNAs.

Supplemental Table 5. The genes information of GO term proton transport and hydrogen transport.

Supplemental Table 6. The information of putative ceRNAs.

Supplemental Table 7. ceRNA target-target and target-mimic pairs.

Supplemental Table 8. lncRNAs cycling in green leaf tip or white leaf base.

Supplemental Table 9. ceRNAs cycling in green leaf tip or white leaf base.

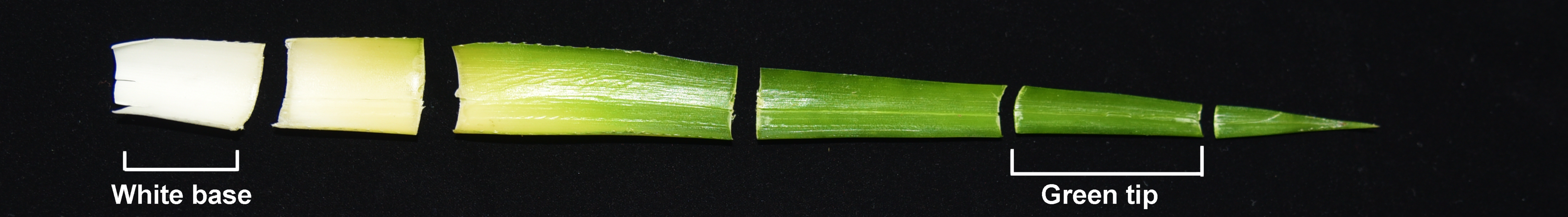
White base
Green tip

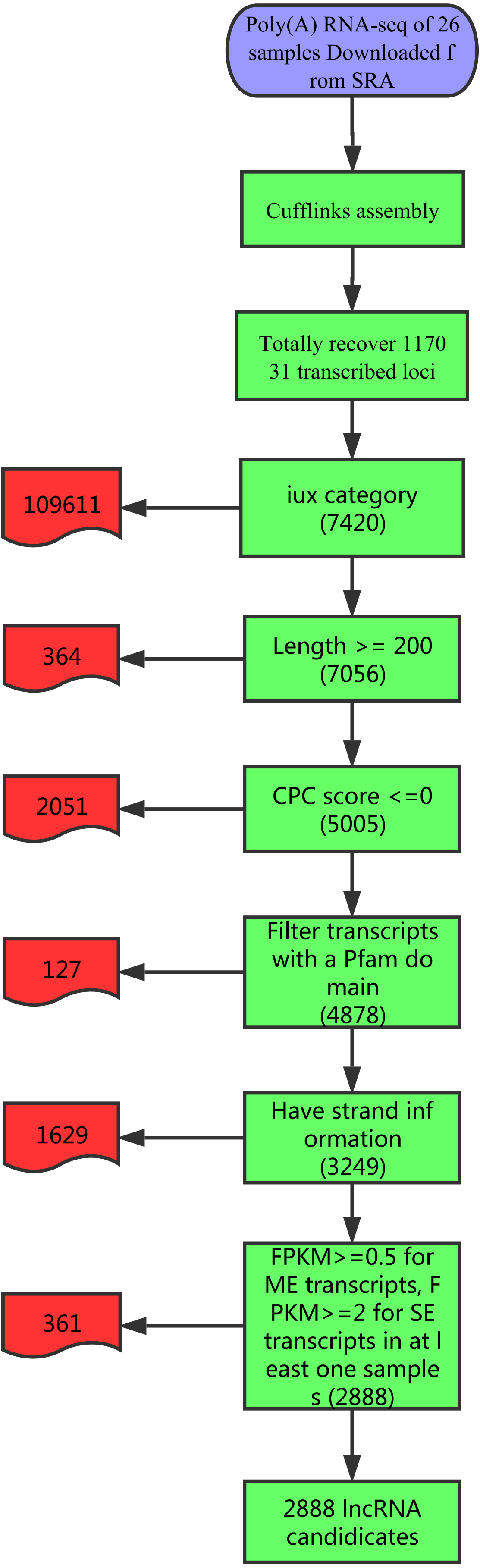
Poly(A) RNA-seq of 26 samples Downloaded f rom SRA
Cufflinks assembly
Totally recover 1170 31 transcribed loci
iux category (7420)
109611
Length >= 200 (7056)
364
CPC score <=0 (5005)
2051
Filter transcripts with a Pfam do main (4878)
127
Have strand inf ormation (3249)
1629
FPKM>=0.5 for ME transcripts, F PKM>=2 for SE transcripts in at l east one sample s (2888)
361
2888 lncRNA candidicates

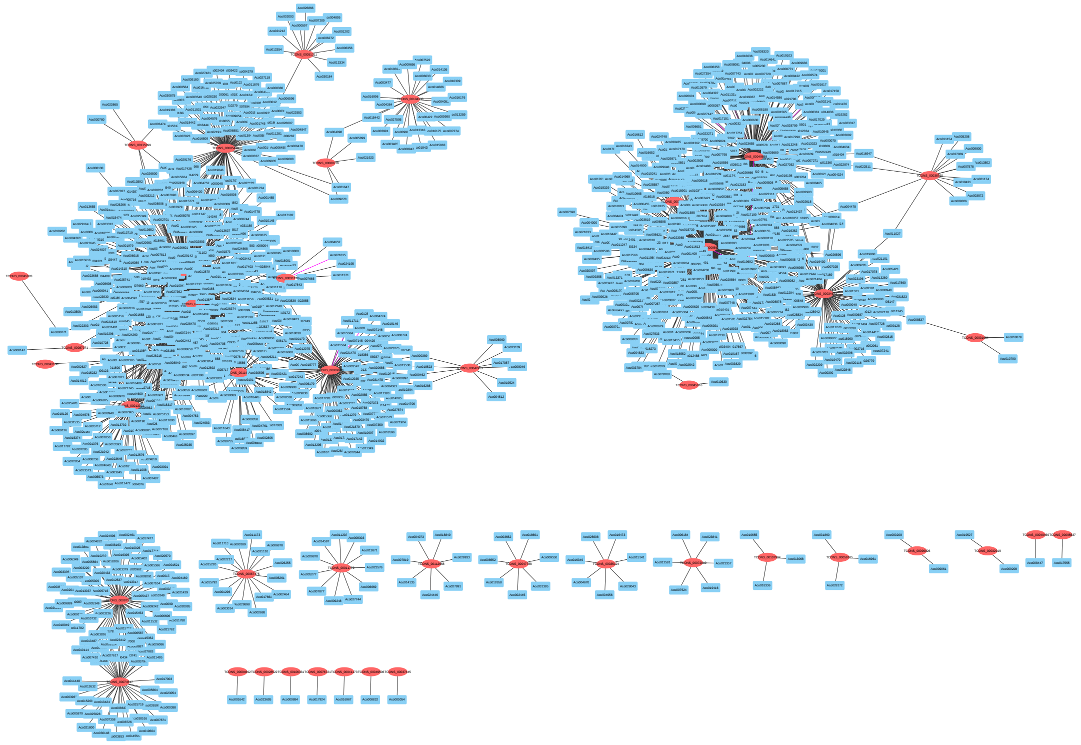

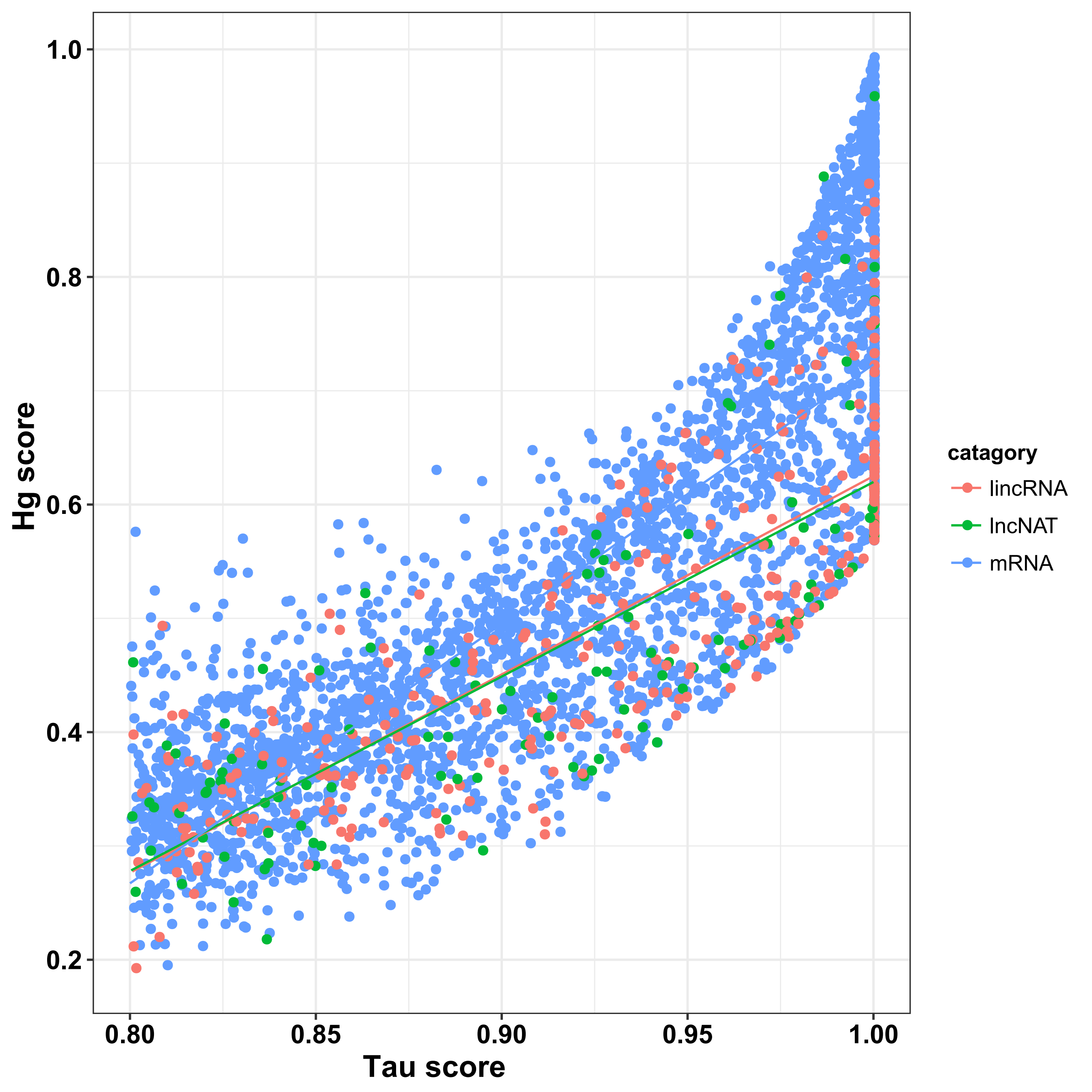

1.0
0.8
0.6
0.4
0.2
Hg score
0.80
0.85
0.90
0.95
1.00
Tau score
catagory
lincRNA
lncNAT
mRNA
